# A meta-analysis of alcohol consumption and thyroid cancer risk

**DOI:** 10.18632/oncotarget.10352

**Published:** 2016-06-30

**Authors:** Xiaofei Wang, Wenli Cheng, Jingdong Li, Jingqiang Zhu

**Affiliations:** ^1^ Department of Thyroid and Breast Surgery, West China Hospital, Sichuan University, Chengdu, China; ^2^ Department of General Surgery, Affiliated Hospital of North Sichuan Medical College, Nanchong, China; ^3^ Department of Otolaryngology-Head and Neck Surgery, Affiliated Hospital of North Sichuan Medical College, Nanchong, China

**Keywords:** thyroid cancer, alcohol consumption, epidemiology, meta-analysis

## Abstract

**Background:**

It is still inconclusive whether alcohol consumption affects the risk of thyroid cancer. We conducted a meta-analysis of available epidemiological data to address this issue.

**Results:**

Compared with nondrinkers, the pooled relative risks (RRs) and corresponding 95% confidential intervals (CIs) of thyroid cancer were 0.80 (95% CI 0.71-0.90) for any drinkers, 0.81 (95% CI 0.70-0.93) for light and 0.71 (95% CI 0.63-0.79) for moderate drinkers. The dose–response analysis suggested that there is no evidence of a dose-risk relationship between alcohol intaking and thyroid cancer risk (*P* = 0.112).

**Methods:**

Eligible studies were identified by searching PubMed and EMbase databases. A total of 24 studies, included 9,990 cases with thyroid cancer, were included in this meta-analysis. We defined light alcohol intake as ≤ one drink/day and moderate as >one drink/day. The summary risk estimates were calculated by the random effects model. A dose-response analysis was also conducted for modeling the dose-risk relation.

**Conclusion:**

This meta-analysis confirmed an inverse association between alcohol consumption and thyroid cancer risk. Further studies are needed to better understand the potential mechanisms underlying this association.

## INTRODUCTION

Thyroid cancer is now recognized as a major public health issue worldwide because of its rapidly increased incidence [1–3]. However, apart from the only well-defined risk factors, such as ionizing radiation exposure especially in childhood [4], benign thyroid conditions [5] and obesity [6], it remains poorly understood on risk factors for thyroid cancer. Alcohol consumption, a highly modifiable behavior, has been acknowledged as a risk factor for cancers of stomach, colorectum, liver, breast and gallbladder [7–11]. However, inconsistent results have been reported on the association between alcohol consumption and thyroid cancer risk. Several epidemiological studies [12–14], including a pooled-analysis [15] of five cohort studies, have suggested an inverse association between alcohol consumption and thyroid cancer risk, while others [16–20], including a pooled-analysis [21] of ten case-control studies, haven't confirmed this association. Recently, Bagnardi et al [11] published a meta-analysis on site-specific cancer risk and alcohol consumption which indicated that light/moderate alcohol intake may decrease the risk of thyroid cancer. However, the result is rough and they didn't consider the potential influence of confounder factors. In addition, the result only based on nine individual studies and many other related studies were not included in their meta-analysis. Therefore, we updated a comprehensive meta-analysis to investigate the possible association of alcohol consumption and thyroid cancer risk.

## RESULTS

### Study characteristics

The literature search strategy identified 154 potentially eligible reports. Finally, 24 studies met the inclusion criteria, including 7 cohort studies [12–14, 16, 17, 32, 33] and 17 case-control studies [18–20, 34–47] (Supplementary Figure S1). The detailed characteristics of these studies were shown in Table 1 and Table 2. Twelve studies were conducted in America, eight in Europe, and four in Asia. Eleven studies reported the overall estimates combined women and men, eight studies only for women; two studies [14, 40] reported respectively the estimates for women, men and the combined women and men. Three studies [18, 33, 38] only reported the estimates separately for different genders (women and men), we considered these reports as separate studies. Therefore, a total of 27 reports derived from 24 studies were included for the meta-analysis. A total of 9,990 cases with thyroid cancer were observed among all these studies. Eleven studies [12–14, 16, 18, 19, 33, 37, 39, 44, 45] reporting at least three categories of alcohol consumption were included into the dose-response analysis. The quality scores ranged from 5 to 9 with a median of 8 for methodological assessment (Supplementary Table S1).

**Table 1:** Characteristics of the case–control studies included in the meta-analysis

Study	Country	Period of enrolment	Gender	Tumor type	No. of cases	No. of controls	Source of control	Variables adjusted for in the regression models
Bandurska 2011 [34]	Europe	1994-2003	WM	TC	297	589	Pop	Matched on age and place of residence.
Choi 2013 [35]	Asia	2010-2011	WM	TC	71	12141	Pop	Age, occupation, household income, marital status, BMI, and smoking.
Franceschi 1989 [36]	Europe	1984-1986	WM	TC	245	411	Hos	Matched on age, sex and geographical area.
Galanti 1997 [37]	Europe	1993-1994	WM	DTC	246	440	Pop	Matched on age, gender, and county of residence.
Guignard 2007 [18]	Europe	1993-1999	WM	DTC	332	412	Pop	Age, ethnic group, and smoking.
Kolonel 1990 [38]	America	1980-1987	WM	DTC	191	441	Pop	Age, gender, ethnicity, education, marital status, smoking, height, weight, occupation, radiation exposure.
Lence-Anta 2014 [39]	America	2000-2011	WM	DTC	203	212	Pop	Age, gender, smoking status, ethnic group, education, number of pregnancies in women, height, and BMI.
Mack 2002 [19]	America	1980-1983	W	TC	292	292	Pop	Age, race, and prior benign thyroid disease.
Menezes 2015 [20]	America	2000-2009	WM	TC	4023	24840	Hos	Age, gender, race, education, marital status, smoking habits, region of residence, and year of diagnosis.
Nagano 2007 [40]	Asia	1970-1986	WM	DTC	362	362	Pop	Age, sex, city, and radiation dose, family history of cancer and past history of goiter or thyroid nodule, smoking.
Prestonmartin 1987 [41]	America	1980-1981	W	TC	110	110	Pop	Matched on age and place of residence.
Riza 2015 [42]	Europe	1990-1993	WM	TC	113	138	Hos	Matched on age, sex, health unit.
Ron 1987 [43]	America	1978-1980	WM	TC	159	285	Pop	Age, sex, prior radiotherapy to the head and neck, thyroid nodules, and goiter.
Rossing 2000 [44]	America	1988-1994	W	PTC	410	574	Pop	Matched on age and county of residence
Stansifer 2015 [45]	America		WM	TC	467	225	Pop	None
Takezaki 1996 [46]	Asia	1988-1993	W	DTC	94	22666	Hos	Age and year of visit.
Xhaard 2014 [47]	Europe	1981-2003	WM	DTC	229	373	Pop	Age, height, BMI, number of pregnancies and menopausal status in women, familial history of thyroid cancer, educational level, medical or therapeutic irradiation of the neck

Abbreviation: W, Women; WM, Women + Men; TC, thyroid cancer; DTC, differentiated thyroid cancer; PTC, papillary thyroid cancer; Pop, population-based; Hos, hospital-based; BMI, body mass index.

**Table 2:** Characteristics of the cohort studies included in the meta-analysis

Author and year of publication	Country and name of the cohort	Period of enrolment	Gender	Tumor type	No. of cases	No. of participates	Duration of follow-up (years)	Variables adjusted for in the regression models
Sungwalee 2013 [32]	Asia, KKCS	1994-2003	W	TC	17	10750	Range: 10-21	Age, occupation, household income, marital status, BMI, smoking.
Sen 2015 [12]	Europe, EPIC	1990	WM	DTC	556	477263	Mean 11	Smoking, education, BMI, physical activity, diabetes, hormone replacement therapy, oral contraceptives, age at menarche, number of full-term pregnancies and menopausal status.
Navarro 2005 [17]	America, NBSS	1992	W	DTC	169	89547	Mean 15.9	Age, education, BMI and smoking.
Meinhold 2009 [14]	America, NIH-AARP	1980	WM	TC	370	490159	Median 7.5	Age, sex, race, education, smoking status, BMI, and family history of cancer.
Kabat 2012 [16]	America, WHI	1995	W	TC	331	159009	Median 12.7	Age, education, smoking, age at first full-term pregnancy, age at menopause, hormone therapy, height (continuous), history of benign thyroid disease.
Allen 2009 [13]	Europe, MWS	1993	W	TC	421	1280296	Mean 7.2	Age, region of residence, socioeconomic status, BMI, smoking, physical activity, use of oral contraceptives, and hormone replacement therapy.
Meinhold 2010 [33]	America, USRT	1983-1984	WM	TC	282	90713	Range: 11-23	Age, smoking status, BMI, number of personal radiographs to the head or neck, cumulative occupational radiation dose, and medical history of benign thyroid conditions.

Abbreviation: W, Women; WM, Women + Men; TC, thyroid cancer; DTC, differentiated thyroid cancer; BMI, body mass index.

Cohort: KKCS, the Khon Kaen Cohort Study; EPIC, the European Prospective Investigation into Cancer and Nutrition; NBSS, Canadian National Breast Screening Study; NIH-AARP, the National Institutes of Health-American Association of Retired Persons Diet and Health Study; WHI, the Women's Health Initiative; MWS, the Million Women Study; USRT, the U.S. Radiologic Technologists Study

### Meta-analysis results

**Figure 1:**
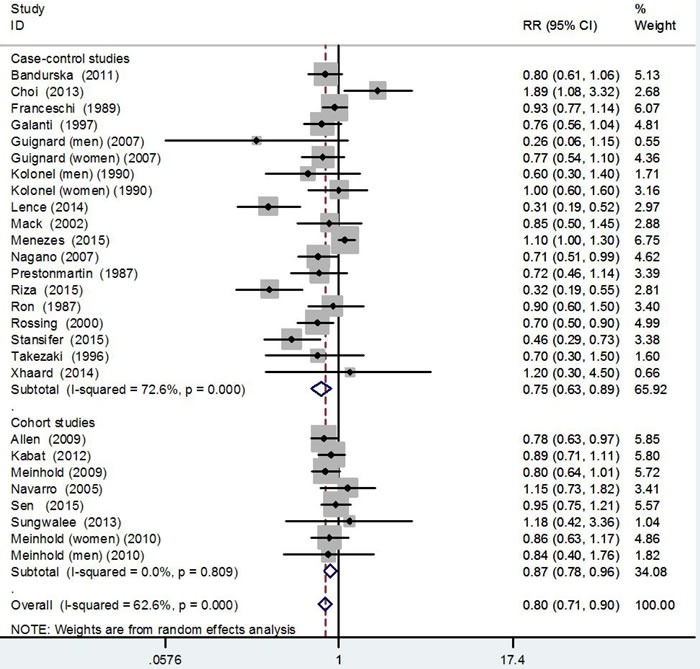
Pooled risk estimates of alcohol drinking for thyroid cancer risk (drinkers *versus* nondrinkers)

Figure 1 shows study-specific and the RR and 95%CI of thyroid cancer risk for alcohol drinking (drinkers *versus* nondrinkers). The pooled RR, based on overall studies, was 0.80 (95% CI 0.71-0.90). The corresponding estimates were 0.87 (95%CI: 0.78-0.96) for cohort studies and 0.75 (95%CI: 0.63-0.89) for case-control studies. Satisfactory homogeneity was found among cohort studies (*I*^2^ = 0%, *P* = 0.809). However, there were significant heterogeneity among case-control studies (*I*^2^ = 72.6%, *P* < 0.01).

**Figure 2:**
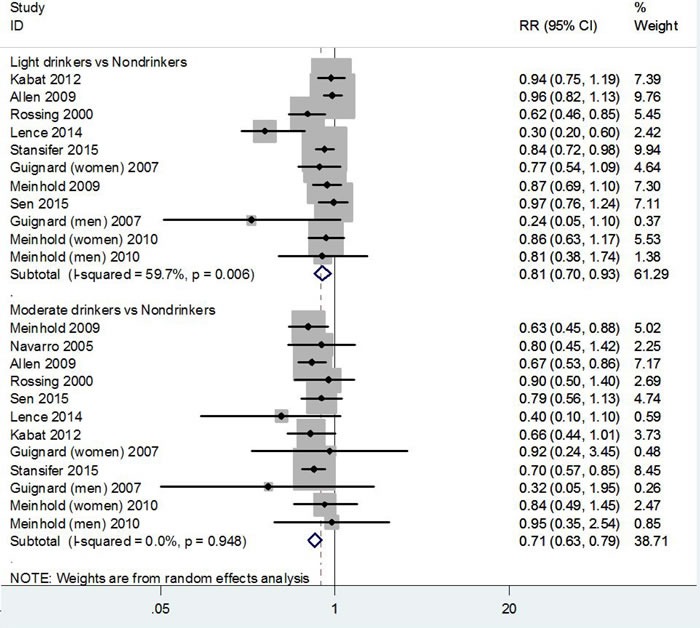
Pooled risk estimates of alcohol drinking for thyroid cancer risk (light or moderate drinkers *versus* nondrinkers)

Figure 2 shows the forest plots of thyroid cancer for light and moderate drinking. Compared to nondrinkers, the pooled estimates were 0.81 (95% CI 0.70-0.93) for light based on nine studies (P for heterogeneity = 0.006) and 0.71 (95% CI 0.63-0.79) for moderate drinkers based on ten studies (P for heterogeneity = 0.948).

Table 3 presents the results of subgroup analyses regarding the association between alcohol consumption and thyroid cancer risk. Because the number of studies that provided data on category of alcohol consumption was small, we only made a subgroup analysis on comparison between any drinkers and nondrinkers. In term of gender, thirteen studies reported combined data for women and men, with a pooled RR of 0.77 (95% CI 0.64-0.94; *P* for heterogeneity < 0.01). Thirteen studies reported data for women, with a pooled RR of 0.81 (95% CI 0.74-0.89; *P* for heterogeneity = 0.886), while only five studies reported data for men, with a pooled RR of 0.77 (95% CI 0.58-1.02; *P* for heterogeneity = 0.599). Considering the geographical area, the pooled RR for thyroid cancer risk was 0.77 (95%CI: 0.65-0.92) in European studies, 0.79 (95%CI: 0.67-0.93) in American studies and 1.01 (95%CI: 0.58-1.75) in Asian studies. Most studies provided multivariate-adjusted results. The adjusted and unadjusted RRs were 0.82 (95%CI: 0.72-0.93) and 0.73 (95%CI: 0.57-0.95) for thyroid cancer risk. Significant heterogeneity was found within the geographical area and whether adjusted for multivariate subgroups (all the *P*-values were < 0.05).

**Table 3:** Pooled RRs of thyroid cancer risk and alcohol consumption (drinkers versus nondrinkers), in strata of selected covariates

	No. of studies/reports	RR (95% CI)	*P* for heterogeneity within subgroup	*P* for heterogeneity between subgroup
Overall	24/27	0.80 (0.71, 0.90)		
Gender ^a^				0.256
Women and Men	13	0.77 (0.64, 0.94)	0.000	
Women	13	0.81 (0.74, 0.89)	0.886	
Men	5	0.77 (0.58, 1.02)	0.599	
Study design				0.937
Cohort	7/8	0.87 (0.78, 0.96)	0.809	
Case-control	17/19	0.75 (0.63, 0.89)	0.000	
Control source ^b^				0.000
Population based	13/15	0.74 (0.62, 0.88)	0.005	
Hospital based	4	0.76 (0.52, 1.11)	0.000	
Geographic area				0.411
America	12/14	0.79 (0.67, 0.93)	0.000	
Europe	8/9	0.77 (0.65, 0.92)	0.021	
Asia	4	1.01 (0.58, 1.75)	0.026	
Publication year				0.447
≥ 2005	15/16	0.79 (0.67, 0.93)	0.000	
< 2005	8/9	0.83 (0.73, 0.94)	0.771	
No. of cases				0.758
≥ 200	16/16	0.80 (0.71, 0.90)	0.000	
< 200	10/11	0.81 (0.60, 1.11)	0.003	
Tumor subtype				0.081
TC	14/15	0.83 (0.72, 0.96)	0.000	
DTC	9/11	0.75 (0.61, 0.93)	0.016	
PTC	1	0.70 (0.50, 0.90)	--	
Quality score				0.014
High (NOS≥ 7)	18/21	0.80 (0.71, 0.91)	0.007	
Low (NOS< 7)	6	0.78 (0.61, 1.01)	0.000	
Adjusted for multivariate				0.217
Yes	18/21	0.82 (0.72, 0.93)	0.000	
No	6	0.73 (0.57, 0.95)	0.013	

a The sum of studies exceeds 24 because five studies reported results for both men and women, separately.

b Among case–control studies only.

RR, relative risk; CI, confidence interval; TC, thyroid cancer; DTC, differentiated thyroid cancer; PTC, papillary thyroid cancer; NOS, Newcastle-Ottawa Scale.

Table 3 also shows significant heterogeneity in subgroup analyses between strata of control source for case-control study, tumor subtype, and NOS quality score (all *P* < 0.10), which may be partly sources of heterogeneity for overall results. However, all of these factors didn't have statistically significant association with the sources of heterogeneity in univariate or multivariate meta-regression analysis (all *P* > 0.10, data not shown). Supplemental Figure S2 shows the results of sensitivity analysis by excluding each study at a time. No study had a significant influence on the overall estimates. The pooled RRs for any drinking varied from 0.78 (when excluding Choi et al [35]) to 0.83(when excluding Lence-Anta et al [39]). The shape of the contour-enhanced funnel plot of studies on the association between alcohol drinking and thyroid cancer risk seemed to be symmetrical, and all the P value of Begg's test were more than 0.05, indicating the absence of publication bias (Supplemental Figure S3).

**Figure 3:**
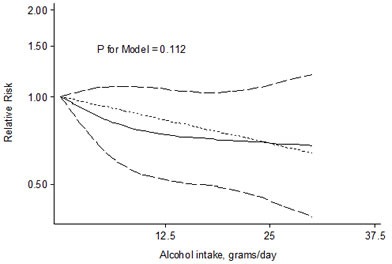
Relative risks (RRs) and the corresponding 95% confidence intervals (CIs) for the dose-response relationship between alcohol drinking (grams per day) and thyroid cancer risk The solid line and the long dash line represent the estimated RRs and their 95% CIs. Short dash line represents the linear relationship.

As shown in Figure 3, the dose-response analysis suggested that there is no evidence of a dose-response relationship between alcohol intaking and thyroid cancer risk (*P* = 0.112). There is no linear relationship for thyroid cancer with every 12.5 g/d alcohol increase (RR = 0.84, 95% CI 0.67-1.04).

## DISCUSSION

In this systematic review, we quantitatively summarized all the available epidemiological evidence on the association between alcohol consumption and thyroid cancer risk and found that any drinking, in relation to nondrinkers, was associated with a 20%, light drinking (≤12.5 g/day of ethanol) with a 19%, and moderate drinking (> 12.5 g/day of ethanol) with a 29% reduced risk for thyroid cancer in present study. However, the association of heavy drinking (> 50 g/day of ethanol) and thyroid cancer risk was unclear due to the lack of quantitative data provided from original studies. The dose-response analysis didn't show a significant dose-response association between alcohol drinking and thyroid cancer risk, but the dose-risk curve, as shown in Figure 3, suggested a reduced trend with increasing alcohol intake in drinkers. This discrepancy between the dose-response analysis and meta-analysis for light and moderate drinkers might be explained by the different methods used and by the different studies included.

In subgroup analyses, we found any drinking, compared with nondrinkers, was associated with a higher pooled risk estimates in cohort studies than that of case-control studies (RR: 0.87 *versus* 0.75). However, among case-control studies, the evidence for an inverse association between alcohol consumption and thyroid cancer risk was limited to the population-based studies. Hospital-based case-control studies did not show a statistical significant association. This may be explained by a higher risk of inappropriate control selection, less control for confounding by smoking or other confounders in hospital-based studies as compared with population-based studies (as shown in Table 1).

Subgroup analysis indicated that the association of alcohol consumption and thyroid cancer risk appeared to be consistent between women and men for any drinkers *versus* nondrinkers. However, we found a stronger association in men compared to women, although there was not statistical difference in terms of men. Possible explanations for these findings might be the higher and more popular alcohol intake in men than women and the limited number of studies reporting data on alcohol intake among men. Of course, the potential differences in alcohol metabolism in women and men maybe have some influence on thyroid cancer risk.

A significant risk difference for thyroid cancer was observed in different geographic areas. The beneficial effect of alcohol intaking on thyroid cancer was found in European (RR = 0.77, 95%CI: 0.65-0.92) and American studies (RR = 0.79, 95%CI: 0.67-0.93), but not in Asian studies (RR = 1.01, 95%CI: 0.58-1.75). This might be due to the different alcohol beverages, different lifestyles and the difference of functional variants in gene involved in alcohol metabolism, such as single nucleotide polymorphisms in ADH1B and ALDH2, among various ethnic groups [48]. The gene polymorphisms involved in alcohol metabolism have been related to the risk of selected cancers [49–51]. However, there is lack of data on their role on thyroid cancer.

Smoking has been suggested as a protective factor for thyroid cancer by several studies [52, 53], which is positively correlated with alcohol drinking. An earlier pooled analysis of ten case-control studies found that there was a significant trend of decreasing thyroid cancer risk for consumption of wine and beer, but that was not maintained after adjustment for smoking [21]. Moreover, thyroid cancer risk was found to be increased among subjects with benign thyroid diseases [5] and obesity [6]. The included studies varied in adjustment for potential confounders. It is impossible to include only articles that adjusted for the same factors. Thus, whenever available, we used multivariate adjusted RRs. The results of adjusted and unadjusted RRs were consistent across strata of whether adjusted for multivariate, which indicated that the inverse association of alcohol consumption and thyroid cancer risk still existed after multivariate adjustment.

There are several potential explanations for the effect of alcohol intake on decreased risk of thyroid cancer. First, light-to-moderate alcohol consumption is associated with enhanced insulin sensitivity and reduced type 2 diabetes [54–56]. Diabetes or obesity has been recognized as a risk for increased thyroid cancer [6, 57, 58]. Second, alcohol intake is associated with reduced prevalence of goitre and solitary thyroid nodules [59], which are important risk factors for thyroid cancer [5]. Some studies have found that alcohol intake have an effect on thyroid volume, thyroid function and the responsiveness of hypothalamic-pituitary-thyroid axis [60–62], which subsequently lead to the change of peripheral thyroid hormone concentrations. However, the influence is elusive with regarding to the peripheral thyroid hormone concentrations on thyroid cancer risk [63–65]. Third, alcohol could also potentially influence thyroid cancer risk by altering sex steroid hormone levels [66–68].

There are some potential limitations to our study. First, marked heterogeneity was detected in several analyses which may reflect differences in study design, study population and adjustment for confounders. Nevertheless, the random-effects models were used to take heterogeneity into account. Meanwhile, subgroup analysis, sensitivity analysis and meta-regression analysis were used to explore the potential heterogeneity in our meta-analysis. Second, former drinkers and sick quitters might have been misclassified into the reference group in some individual studies and thus a decreased effect has been estimated. We could not address this issue because the majority of the studies did not report separate estimates between former drinkers and lifelong never drinkers. Third, most studies (13/24) collected information by self-reporting questionnaires, which might lead to information bias. However, several studies have found satisfactory correlation coefficients for validity and reproducibility of self-reported alcohol drinking in various populations [69].

Notwithstanding the limitations discussed above, this meta-analysis includes the most comprehensive information with a large sample size on alcohol consumption and thyroid cancer risk to date, and confirms an inverse association of alcohol consumption for thyroid cancer risk. Further studies are needed to better understand the potential mechanisms underlying this association.

## MATERIALS AND METHODS

### Search strategy and inclusion criteria

We identified all relevant case-control and cohort studies published in English by searching the two databases (PubMed and EMbase) from the beginning of indexing to August 2015, using the following terms: (alcohol OR ethanol) AND (thyroid tumor OR thyroid cancer OR thyroid carcinoma OR thyroid neoplasm) AND (cohort OR prospective OR case-control), following the Meta-analysis Of Observational Studies in Epidemiology (MOOSE) guidelines [22]. Two authors (XF Wang and WL Cheng) independently assessed and identified potentially relevant articles, and reviewed the references list in the articles and associative reviews to identify additional studies. The inclusion criteria were as follows: (1) cohort study or case-control study published as original articles; (2) evaluated the association of alcohol consumption and thyroid cancer incidence in general population; (3) provided the relative risk (RR)/odds ratio (OR)/hazard ratio (HR) and the corresponding 95% confidence interval (CI) or sufficient information to enable calculation. Abstracts or unpublished reports were not considered for inclusion in the meta-analysis.

### Data extraction and quality assessment

Two authors (Wang XF and WL Cheng) independently extracted data from each original article using a pretested form. The extracted data were composed of first author, year of publication, country, gender, study design, period of enrollment and source of controls (case-control studies), follow-up time (cohort studies), sample size (numbers of cases, controls or non-cases or cohort size), variables adjusted for estimates, risk estimates (Ors, RRs or HRs) for alcohol consumption levels and the corresponding 95% CIs. All risk estimates results were expressed as RRs. The ORs and HRs approximate to the RRs because of the low incidence of thyroid cancer in general population.

Two authors (Wang XF and Li JD) independently assessed the quality of included studies according to the Newcastle-Ottawa Scale (NOS) [23]. The NOS includes three broad perspectives: selection (four items), comparability (two items) and exposure/outcome (three items). The full score was 9 points, and the study with awarded points ≥7 was defined as a high quality study. Disagreements were discussed and resolved with consensus.

### Statistical analyses

The expression of alcohol intake was various in different studies; therefore, we used grams of ethanol per day as a standard measure and defined one drink as 12.5 g of ethanol, 1 ml of alcohol as 0.8 g and 1 ounce as 28 g. Nondrinkers drinkers were defined as the reference category. Since the highest category of alcohol exposure was set at > 1 drinks/day in the majority of studies, thus we divided the alcohol drinker into two levels: light drinker defined as ≤1 drink/day (≤12.5 g/day of ethanol) and moderate as >1 drinks/day (>12.5 g/day of ethanol). When a study provided a series of categories of alcohol consumption, we used the midpoint of each category as the corresponding exposure dose. For the upper open-ended category, the exposure dose was calculated as 1.2 times its lower bound [24]. When the estimates for drinkers versus nondrinkers was not presented in a study, we combined the corresponding risk estimates associated with different alcohol exposure categories using the method proposed by Hamling et al [25] when possible. Similar methods were adopted for light and moderate drinker when more than one exposure categories fell in one of these levels. When a study didn't used nondrinkers as a reference category, the RRs and 95% CI were recalculated using the nondrinkers as reference by the method proposed by Orsini et al [26].

The multivariate-adjusted RRs were used for the meta-analyses; when they are unavailable, the unadjusted RRs were calculated from original data. The pooled RRs with 95% CIs were calculated by random effects models [27] for the association between drinkers and nondrinkers. Heterogeneity among articles was quantitatively assessed using the Q test and *I*^2^ statistic [28]. A significant heterogeneity was defined as the *I*^2^ more than 50% or Q-test reporting a *P* value < 0.1. To explore the potential sources of heterogeneity among studies, we conducted meta-regression analysis and subgroup analyses in strata of gender, study design, control source for case-control study, geographic area, publication year, number of cases, tumor subtype, NOS quality score and whether adjusted for multivariate. Sensitivity analyses were also performed by excluding each study at a time to clarify the influence of each study on the overall estimates. Publication bias was assessed by Begg's rank correlation test [29] and the contour-enhanced funnel plot [30].

A potential nonlinear dose-response relationship between alcohol consumption and thyroid cancer risk were conducted based on the natural logarithm of the RR for each study with at least three quantitative categories of exposure using the methods described by Orisini [31]. The P value for nonlinearity was calculated by testing the null hypothesis that the coefficient of the second spline was equal to zero.

A *P* value less than 0.05 was considered to be statistically significant. All *P* values were two-tailed. All calculations were performed using Stata 11.0 (Stata Corporation, College Station, TX).

## SUPPLEMENTARY MATERIALS


