# Supplementary material for: A meta-analysis of alcohol consumption and thyroid cancer risk
Source: Oncotarget. 2016 Jun 30;7(34):55912–23. doi: 10.18632/oncotarget.10352 (PMC5342461; doi:10.18632/oncotarget.10352)
Supplement: Supplementary file 1 [file oncotarget-07-55912-s001.pdf]

A meta-analysis of alcohol consumption and thyroid cancer risk

Supplementary Material

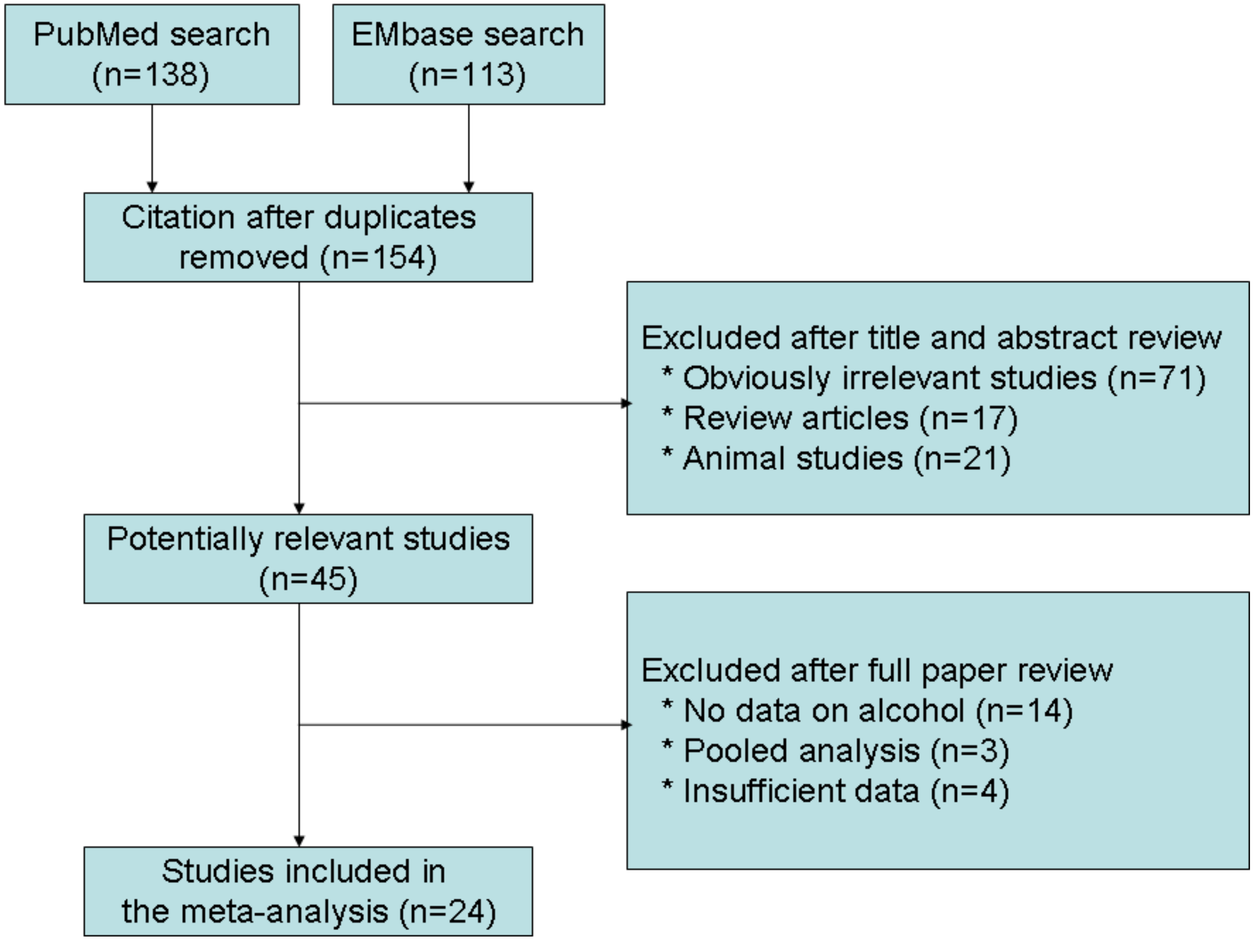

Figure S1. Flowchart for search and selection of studies for inclusion in this meta-analysis.

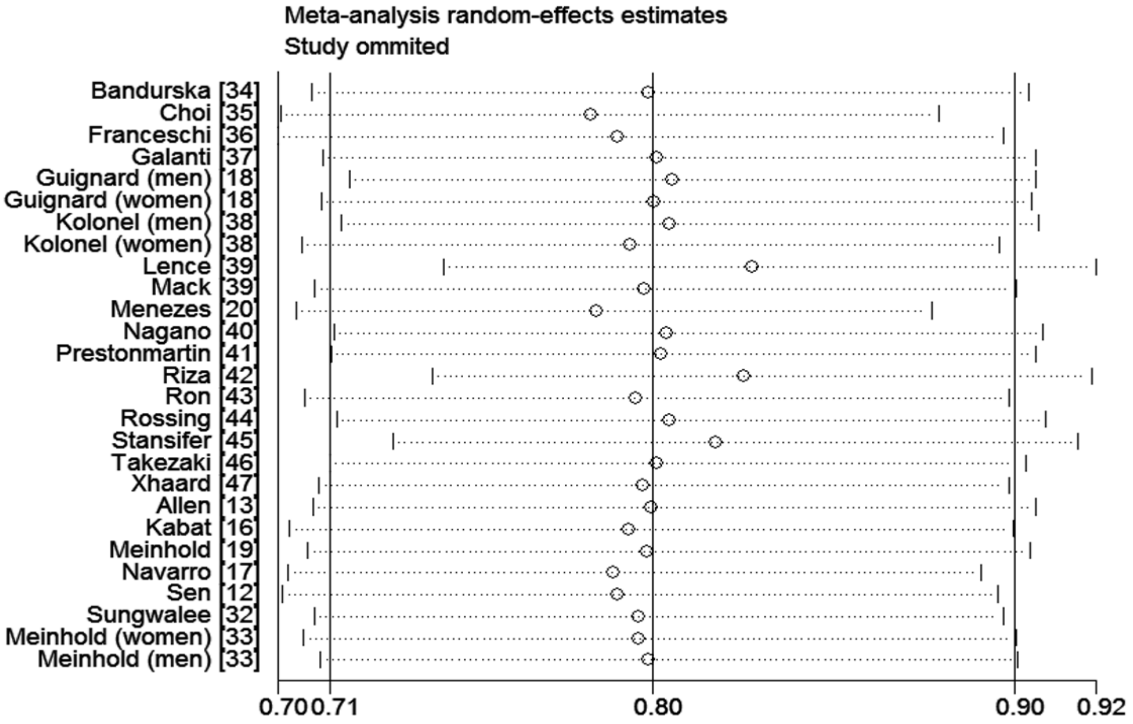

Figure S2. Sensitivity analysis by sequential removing a specific study.

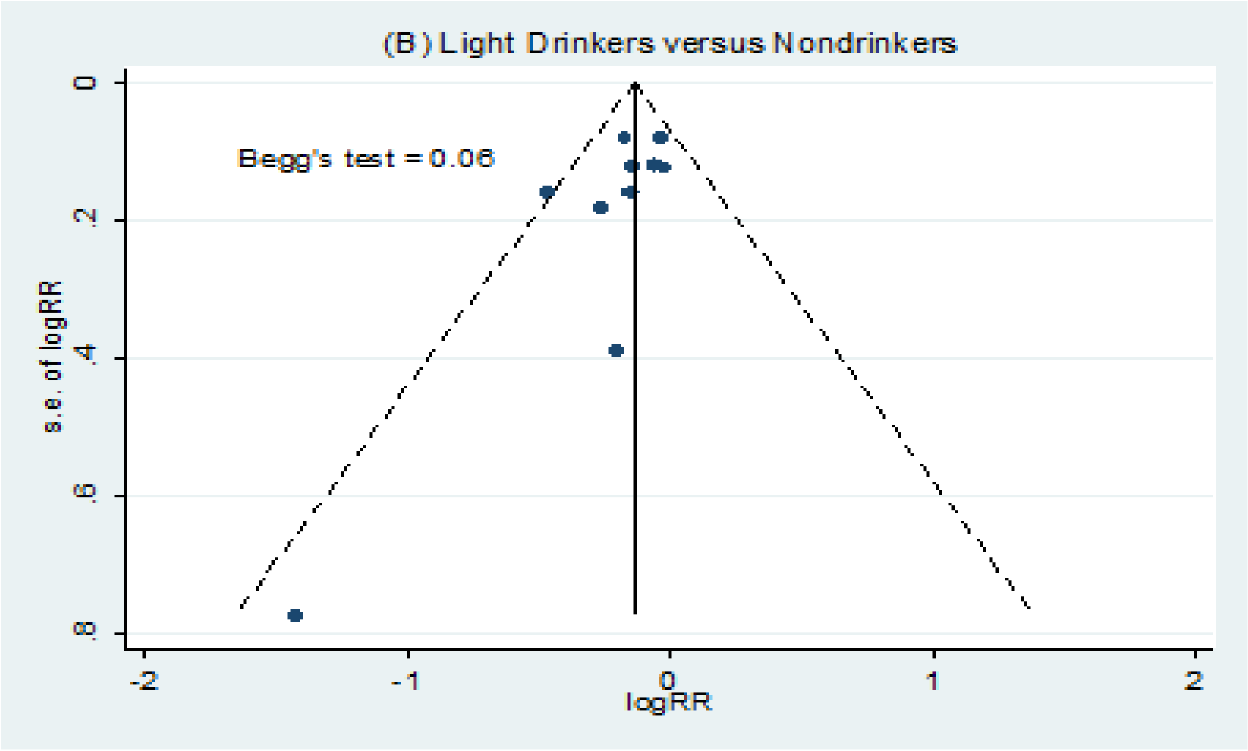

Figure S3. Publication bias by funnel plot of log relative risk (RR) versus corresponding standard error and the Begg's test.

Supplementary Table S1

Table S1. Newcastle-Ottawa Quality Assessment Scale for Studies Included in the Meta-analysis

Table S1A. Methodologic quality of cohort studies included in the meta-analysis

Author and year of publication	Selection				Comparability	Outcome			Total quality Scores (★)
	Representativeness of the exposed cohort	Selection of the non-exposed cohort	Ascertainment of exposure	Incident events		Assessment of outcome	Length of follow-up	Adequacy of follow-up of cohort	
Sungwalee 2013 [32]	A	A	C	A	A	A	A	A	7
Sen 2015 [12]	A	A	C	A	AB	B	A	A	8
Navarro 2005 [17]	A	A	C	A	AB	B	A	A	8
Meinhold 2009 [14]	A	A	C	A	AB	A	B	A	7
Kabat 2012 [16]	A	A	C	A	AB	A	A	A	8
Allen 2009 [13]	A	A	C	A	AB	B	B	A	7
Meinhold 2010 [33]	A	A	C	A	AB	A	A	A	8

Selection: (1) Representativeness of the exposed cohort: A, truly representative of the average patient with thyroid cancer ★; B, somewhat representative of the average patient with thyroid cancer ★; C, selected group; and D, no description of the derivation of the cohort. **(2) Selection of the non-exposed cohort:** A, drawn from the same community as the exposed cohort ★; B, drawn from a different source; and C, no description of the derivation of the non-exposed cohort. **(3) Ascertainment of exposure:** A, secure record ★; B, interviewer-administered questionnaire ★; C, self-administered questionnaire; and D, no description. **(4) Demonstration that outcome of interest was not present at start of study:** A, yes ★; B, no.

Comparability: (5) Comparability of cohorts on the basis of the design or analysis: A, study controls for age, gender ★; B, study controls for additional risk factors (drinking, BMI, diabetes, et al)★.

Outcome: (6) Assessment of outcome: A, confirmed by medical records, ★; B, identified through ICD codes on database records ★; C, self-report; and D, no description. **(7) Was follow-up long enough for outcomes to occur: (mean of follow-up time more than 10 year)** A, yes ★; B, no; C, no statement. **(8) Adequacy of follow-up of cohorts:** A, complete follow-up all subjects accounted for ★; B, subjects lost to follow-up unlikely to introduce bias (small number lost), follow-up rate higher than 90%,or description provided of those lost ★; C, follow-up rate 90% or lower and no description of those lost; and D, no statement.

Table S1B. Methodologic quality of case-control studies included in the meta-analysis

Authors and year of publication	Selection				Comparability	Exposure			Total quality Scores (★)
	Adequate definition of cases	Representativeness of cases	Selection of controls	Definition of controls		Asecertainment of exposure	Same method of ascertainment for cases and controls	Non-response rate	
Bandurska 2011 [34]	B	A	A	A	A	C	A	B	5
Choi 2013 [35]	B	A	A	A	AB	B	A	A	8
Franceschi 1989 [36]	A	A	B	A	A	C	A	B	5
Galanti 1997 [37]	A	A	A	A	A	C	A	B	6
Guignard 2007 [18]	A	A	A	A	AB	B	A	A	9
Kolonel 1990 [38]	A	A	A	A	AB	B	A	A	9
Lence-Anta 2014 [39]	B	A	A	A	AB	B	A	A	8
Mack 2002 [19]	B	A	A	A	AB	B	A	A	8
Menezes 2015 [20]	B	A	B	A	AB	C	A	B	5
Nagano 2007 [40]	A	A	A	A	AB	B	A	A	9
Prestonmartin 1987 [41]	B	A	A	A	A	B	A	A	8
Riza 2015 [42]	A	A	B	A	A	B	A	B	6
Ron 1987 [43]	A	A	A	A	AB	B	A	A	9
Rossing 2000 [44]	B	A	A	A	AB	B	A	C	7

Stansifer 2015 [45]	A	A	A	A	AB	C	A	B	7
Takezaki 1996 [46]	A	A	B	A	AB	C	A	B	6
Xhaard 2014 [47]	A	A	A	A	AB	B	A	B	8

Selection: (1) Is the case definition adequate? A, yes, reference to medical/hospital records ★; B, yes, record linkage (e.g. ICD codes in database) or self-report; C, no description. **(2) Representativeness of the cases.** A, consecutive or obviously representative series of cases ★; B, potential for selection biases or not stated. **(3) Selection of Controls.** A, community controls ★; B, hospital controls; C no description. **(4) Definition of Controls.** A, no history of disease ★; B, no description of history of disease.

Comparability: (5) Comparability of cases and controls on the basis of the design or analysis. A, study controls for age, gender ★; B, study controls for any additional factor ((drinking, BMI, diabetes, et al)) ★.

Exposure: (6) Ascertainment of exposure. A, secure record ★; B, interviewer-administered questionnaire ★; C, self-administered questionnaire; and D, no description. **(7) Same method of ascertainment for cases and controls.** A, yes ★; B, no. **(8) Non-Response rate.** A, same rate for both groups ★; B, non respondents described; C, rate different and no designation
